# Anesthetic management of a pediatric patient with Freeman-Sheldon syndrome undergoing atrial septal defect closure: a case report

**DOI:** 10.1186/s40981-023-00633-9

**Published:** 2023-07-13

**Authors:** Kyosuke Takahashi, Kotaro Sakurai, Izumi Hamaya

**Affiliations:** 1 Department of Anesthesiology and Critical Care Medicine, Jichi Medical University Saitama Medical Center, 1-847 Amanumacho, Saitama, 330-0834 Japan; 2 Department of Anesthesia and Critical Care, Kawasaki Municipal Hospital, 12-1 Shinkawa-Dori, Kawasaki, 210-0013 Japan; 3 Department of Anesthesiology, Kyoto University Hospital, 54 Shogoin-Kawahara-Cho, Kyoto, 606-8507 Japan; 4 Department of Cardiac Anesthesia, Saitama Children’s Medical Center, 1-2 Shin-Toshin, Saitama, 330-8777 Japan

**Keywords:** Freeman-Sheldon syndrome, Child, Cardiac surgery, Difficult airway

## Abstract

**Background:**

Freeman-Sheldon syndrome (FSS) is a rare disorder characterized by specific deformities of the extremities and face. There have been no reports of open-heart surgery in pediatric patients with FSS.

**Case presentation:**

We present the case of an 8-year-old girl with FSS who underwent atrial septal defect closure. Tracheal intubation was uncomplicated, although the patient had microstomia. Inhalational anesthetics and dopamine antagonists were avoided intraoperatively and perioperatively. We chose dexmedetomidine as an adjuvant for postoperative pain management contributing to adequate analgesia and early extubation without causing respiratory depression.

**Conclusions:**

Anesthetic management of FSS requires consideration for airway management and prevention of malignant hyperthermia and respiratory complications. We successfully managed the case avoiding the use of malignant hyperthermia-triggering drugs.

## Background

Freeman-Sheldon syndrome (FSS) is a rare congenital disorder that is inherited in an autosomal dominant pattern [1]. It is characterized by a peculiar facial appearance with microstomia and pouting lips described as “whistling face,” deformities of the fingers and feet, and progressive myopathy [2].

Nearly every individual with FSS requires multiple surgical procedures, and anesthetic management is challenging. In addition to difficult airways due to unique facial features, malignant hyperthermia and muscle rigidity have also been reported in patients receiving malignant hyperthermia-triggering anesthesia [3–8]. To date, there have been no reports of open-heart surgery in pediatric patients with FSS. Herein, we present a pediatric case of FSS that underwent atrial septal defect (ASD) closure. Written informed consent for publication including images was obtained from the patient and her parents. This manuscript adheres to the CARE guidelines.

## Case presentation

An 8-year-old girl, 125-cm tall and weighing 21.6 kg, was diagnosed with FSS in infancy due to contractures of the extremities and a peculiar facial appearance. At the age of 2 years, she underwent multiple surgeries for clubfoot that were performed under general anesthesia. At that time, mask ventilation was easy, and she was intubated using Pentax Airway Scope (optical video laryngoscope) without difficulty. ASD was diagnosed when she presented to the hospital with a complaint of left thorax deformity at the age of 7 years. Transesophageal echocardiography (TEE) revealed a type 2 ASD (maximum diameter, 10 mm) and enlargement of the right heart. General anesthesia for TEE was complicated by difficult intubation. The anesthesia induction was started with sevoflurane and nitrous oxide. Subsequent to a IV-line insertion, propofol and remifentanil were used for maintenance. Although mask ventilation was easy, the Cormack-Lehane grade was 4 with a Macintosh laryngoscope. Airway Scope was used for tracheal intubation.

**Fig. 1:**
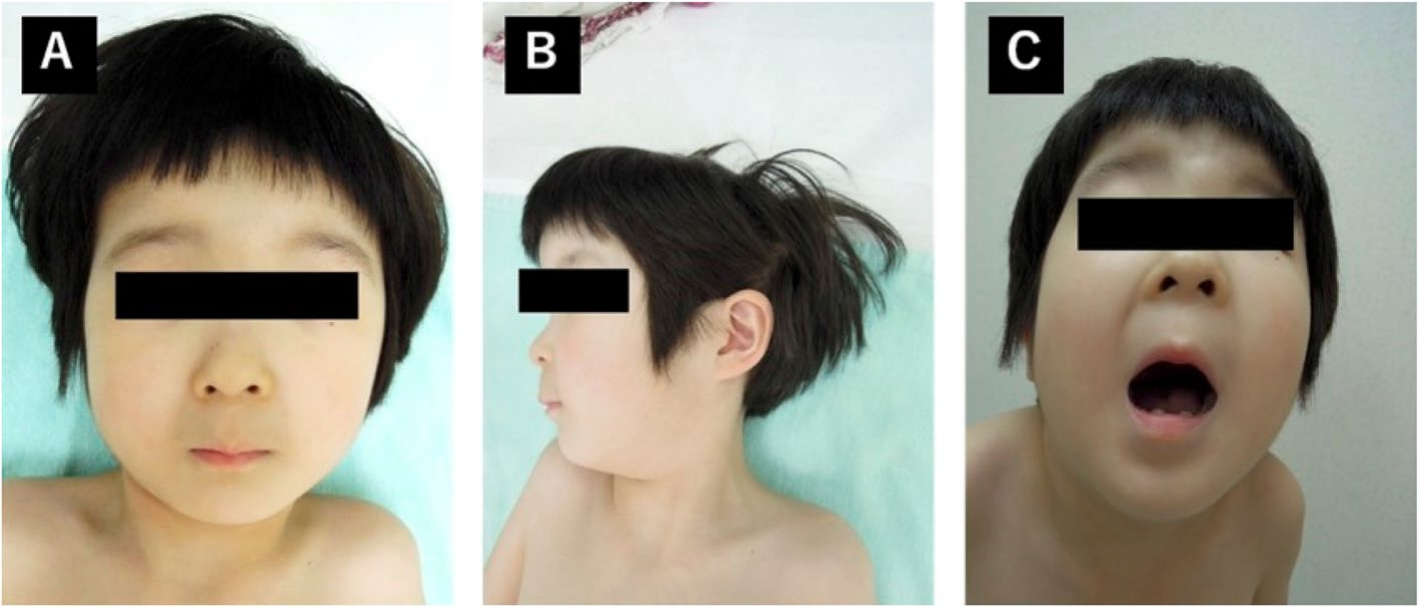
**A** Frontal and **B** lateral views of the patient, showing microstomia, whistling facial expression, H-shaped chin dimple, and deep nasolabial folds. **C** There was no restriction in mouth opening

**Fig. 2:**
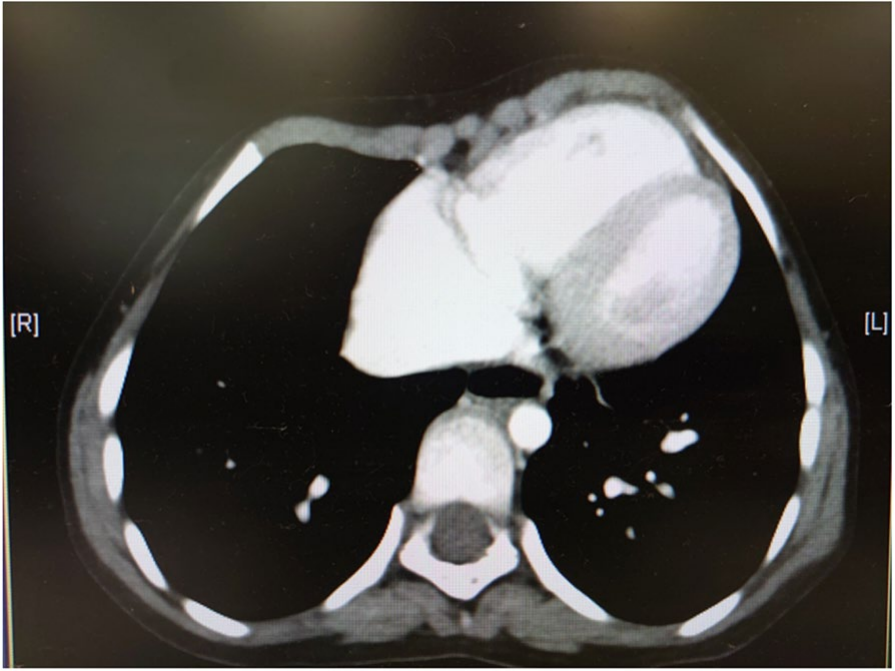
Computed tomography scan of the thorax. Deformity of the rib cage is seen

ASD closure was planned 1 year after the TEE examination. Physical examination revealed microstomia, whistling facial expression, H-shaped chin dimple, and deep nasolabial folds (Fig. 1A, B). There were no restrictions on mouth opening (Fig. 1C) and neck retroflection. Protrusion of the left rib cage was noted. Computed tomography revealed a rib cage deformity (Fig. 2), but the heart and lungs were not compressed.

Total intravenous anesthesia was planned for the surgery. Venous access was not difficult, and a 22-G catheter was inserted. Anesthesia was induced with fentanyl 50 μg, propofol 40 mg, rocuronium 20 mg, and atropine sulfate 0.2 mg. Mask ventilation was easy; Macintosh laryngoscopy revealed a Cormack-Lehane grade II, and a 6.0-mm tracheal tube was easily inserted. Although Airway Scope was prepared in anticipation of difficult intubation, it was not needed. Anesthesia was maintained with remifentanil 1 mcg・kg^−1^・min^−1^ and propofol 6 mg・kg^−1^・h^−1^. These doses were adjusted according to hemodynamic changes and aimed for a bispectral index between 40 and 60. Surgery was performed via median sternotomy, and direct closure of the ASD was performed after cardiopulmonary bypass was established. After weaning from cardiopulmonary bypass, dexmedetomidine was started at 0.7 mcg・kg^−1^・h^−1^ for postoperative analgesia.

Prior to extubation, sugammadex 4 mg・kg^−1^ was administered. The patient was extubated in the operating room without complications and transferred to the intensive care unit. Acetaminophen 15 mg/kg was administered by an intravenous infusion every 6 h for postoperative analgesia, and dexmedetomidine infusion was continued until chest drain removal on the third postoperative day. The face pain scale score had been < 3 throughout the course. The postoperative course was uneventful, and the patient was discharged on the 11th postoperative day.

## Discussion

FSS is a group of diseases first described in 1938 [4] and is classified as distal arthrogryposis type 2A [5]. Clinically, microstomia with pursed lips (whistling face), bent fingers with ulnar deviations, and clubfoot are characteristic and diagnosed by ultrasound during prenatal period or physical examination at birth [4]. Other features include strabismus and scoliosis, which require multiple surgical interventions under general anesthesia during childhood [5], while no literature mentions congenital heart disease in FSS. Previous studies have reported difficult airways, malignant hyperthermia during general anesthesia, and postoperative respiratory complications. Therefore, handling these issues is the essence of management.

Difficulty in airway management is attributed to the multiple anatomical deformities of FSS. Fibrosis of the orbicularis oris muscle and a fibrous band along the vermilion border of the lower lip result in microstomia and pursed lips. Hypoplasia of the mandible and high-arched palate leads to narrowing of the oral cavity. Muscle contractures cause a short and relatively immobile neck. These features make direct laryngoscopy and tracheal intubation difficult [2].

Various approaches to managing difficult airways in FSS have been reported [2, 6–8]. Fiber-optic intubation under mild sedation can be safely performed in adult patients [6], but it is often difficult in pediatric patients who are unable to cooperate with the procedure. There are reports of nasotracheal intubation using a fiber-optic bronchoscope with spontaneous breathing [7] and using a laryngeal mask in minor surgery [8]. In this case, mask ventilation was easy in the previous induction of general anesthesia, and we chose intravenous induction. Contrary to previous reports, the reason for the ease of intubation in this case is unclear. It is possible that the influence of microstomia on airway opening has become relatively less through growth with age.

Congenital myopathy is considered a risk factor for malignant hyperthermia [9]. Malignant hyperthermia in patients with FSS triggered by halothane and succinylcholine has been reported [5, 9]. Although there is a report of successful management of pediatric FSS patients with induction and maintenance using sevoflurane [6], the present case was managed with total intravenous anesthesia for safety. Neuroleptic malignant syndrome is another complication that should be prevented. Neuroleptic malignant syndrome is a potentially lethal drug reaction associated with dopamine antagonists. Neuroleptic malignant syndrome in infants with FSS induced by metoclopramide has been reported [10]. In addition to metoclopramide, chlorpromazine, which is sometimes used as a vasodilator in open-heart surgery in children, should be avoided as it acts as a dopamine antagonist.

FSS is a risk factor for postoperative respiratory complications due to decreased respiratory muscle mass caused by myopathy and restrictive disorders caused by thorax deformation [5, 6]. Therefore, it is necessary to avoid respiratory depression while providing adequate postoperative analgesia [2]. Dexmedetomidine is an option for perioperative pain relief. Dexmedetomidine reduces opioid consumption during the perioperative period [11]. Moreover, it suppresses the response to surgical stress [12], prevents tachyarrhythmia, and shortens the duration of postoperative mechanical ventilation in pediatric cardiac surgery [13]. Considering these, dexmedetomidine can be helpful as an adjunctive intra- and postoperative analgesic for patients in whom inhalational anesthesia cannot be used, as in this case.

Hemodynamic effects of dexmedetomidine should be noted with its use in pediatric cardiac surgery. The adverse effects are dose dependent, and repeated large boluses of 2–3 mcg・kg^−1^ can trigger hypotension. Bradycardia is common, and heart rate is expected to decrease up to 30% from baseline. Although hemodynamic compromise is modest in most cases, there is a report of extreme bradycardia in an infant receiving dexmedetomidine and digoxin [14]. Another report described cardiac arrest of two neonates treated with co-administration of amiodarone and dexmedetomidine [15]. Hence, dexmedetomidine should be used with caution, particularly when administered with anti-arrhythmic agents.

In conclusion, anesthesia was successfully managed for open-heart surgery in this pediatric patient with FSS. It is crucial to prepare for airway difficulty and avoid malignant hyperthermia-inducing drugs. Dexmedetomidine can be a viable option in terms of postoperative analgesia and the prevention of respiratory complications.

## Data Availability

Not applicable.

## Abbreviations

ASD: Atrial septal defect
FSS: Freeman-Sheldon syndrome
TEE: Transesophageal echocardiography
